# The hidden burden: incidentalomas detected on abdominopelvic computed tomography in bariatric surgery candidates – a single-center experience

**DOI:** 10.25122/jml-2025-0174

**Published:** 2025-11

**Authors:** Mihaela Toader, Costin Chirica, Liliana Gheorghe, Sabina-Ioana Chirica, Danisia Haba, Ana-Maria Buburuz, Mădălina Maxim, Daniel Vasile Timofte

**Affiliations:** 1 Doctoral School, Grigore T. Popa University of Medicine and Pharmacy, Iasi, Romania; 2 Department of Radiology, Grigore T. Popa University of Medicine and Pharmacy, Iasi, Romania; 3 Department of Oral and Maxillofacial Surgery, Grigore T. Popa University of Medicine and Pharmacy, Iasi, Romania; 4 Cardiology Clinic, St. Spiridon County Clinical Emergency Hospital, Iasi, Romania; 5 1st Medical Department, Grigore T. Popa University of Medicine and Pharmacy, Iasi, Romania; 6 Department of Surgery, St. Spiridon County Clinical Emergency Hospital, Iasi, Romania; 7 Department of Surgery, Grigore T. Popa University of Medicine and Pharmacy, Iasi, Romania

**Keywords:** incidentaloma, obesity, bariatric surgery, computed tomography

## Abstract

Bariatric surgery (BS) is currently one of the major breakthroughs in the management of patients with morbid obesity, showing outstanding long-term results. While an abdominopelvic computed tomography (CT) aims to analyze fat distribution and anatomical integrity during a preoperative evaluation, incidentalomas are unexpected tumors or abnormalities discovered by chance on imaging. The purpose of this study was to present our institution's experience with incidental findings detected during preoperative CT evaluations in patients who are candidates for BS. In our retrospective, observational study, we analyzed preoperative CT images from 131 patients eligible for BS and selected only those diagnosed with incidentalomas. Among the 29 obese patients (89.7% female; mean age, 39.0 ± 11.6 years; mean BMI, 38.5 ± 4.6 kg/m^2^) who were candidates for BS and had incidentalomas detected on a preoperative CT, a total of 64 lesions were identified, with a mean of 2.2 ± 1.3 per patient. A significant positive correlation was observed between age and the number of incidentalomas found (r = 0.52, *P* = 0.004), with hepatic involvement being the most common type (31.2%). In summary, our study highlights that incidentalomas are not mere chance occurrences but rather a valuable and common finding in this patient population. Their discovery can have a significant impact on surgical planning, potentially requiring a modified approach, further investigation, or even contraindicating the planned BS. The findings emphasize the importance of thorough preoperative CT evaluations for BS candidates.

## INTRODUCTION

Obesity remains a major public health concern with an alarming prevalence and an exponential increase over the last decade. The World Health Organization's 2016 global estimate documented approximately 650 million adults affected by obesity. However, emerging evidence from contemporary modeling studies – including large-scale epidemiological analyses – suggests that this figure has since risen significantly, potentially surpassing the threshold of 1 billion individuals worldwide [1-3]. In the United States, the prevalence of obesity increased from 30.5% to 42.4% between 2000 and 2018, and the prevalence of morbid obesity increased from 4.7% to 9.2%. This upward trend is also observed in other developed and developing countries, reflecting global changes in lifestyle, diet, and physical activity [4].

In addition, this complex and multifactorial condition is not just a matter of excess weight, but also a significant risk factor for several severe comorbidities, including type 2 diabetes, hypertension (HT), dyslipidemia (DL), coronary heart disease, and certain types of cancer. Also, excess body weight is associated with a decline in quality of life, mental health problems such as anxiety and depression, and a reduced life expectancy compared to the general population [5,6].

**Figure 1:**
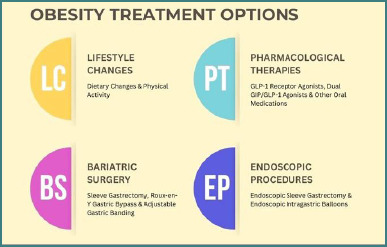
Overview of treatment approaches for overweight and obesity

In the context of this global epidemic, various intervention strategies have been developed, ranging from lifestyle changes and pharmacological therapies to surgical interventions and endoscopic procedures, aimed at reducing body weight and mitigating the risks associated with obesity (Figure 1) [7].

However, in cases of morbid obesity, where the body mass index (BMI) exceeds 40 kg/m^2^ or 35 kg/m^2^ in the presence of significant comorbidities, bariatric surgery (BS) has proven to be an effective and sustainable therapeutic option, capable of inducing substantial weight loss and improving or resolving many of the associated conditions [8, 9].

Consequently, BS is currently one of the major breakthroughs in the management of patients with morbid obesity, showing outstanding long-term results. The various types of obesity surgery, such as gastric bypass or Roux-en-Y bypass (RYGB) and sleeve gastrectomy (SG), require a case-by-case assessment and decision based on well-documented clinical and paraclinical data [10,11].

Therefore, performing a surgical procedure for this purpose requires thorough planning and often an abdominopelvic computed tomography (CT) scan for an accurate overview. In most cases, choosing other imaging modalities, such as ultrasound, does not provide sufficient information for pre-BS assessment, and CT is the preferred investigation. Thus, this practice can be justified by the need for a thorough assessment of the anatomy and potential pathologies that could complicate the bariatric procedure [12].

Accordingly, with the evolution of various imaging techniques, especially CT, a number of associated asymptomatic disorders and lesions, called incidentalomas, may be discovered in patients who are candidates for BS. While an abdominopelvic CT aims to analyze fat distribution and anatomical integrity during a preoperative evaluation in patients with morbid obesity, incidentalomas are often discovered by chance. Thus, in this case, incidentalomas are unexpected and often unsuspected findings identified during imaging investigations initially performed for other clinical indications. Identification of these findings can have a major impact on both operative and postoperative outcomes [13-15].

Incidentalomas are not just simple observations, but also valuable insights into patient outcomes [16-18]. For example, a CT investigation can identify conditions such as gallstones, pancreatitis, splenomegaly, or other abnormalities that may require a modified surgical approach or even contraindicate BS. Moreover, the presence of cholecystitis or pancreatitis may require the resolution of these issues prior to BS to reduce the risk of postoperative complications [19].

Nevertheless, morbidly obese patients already have an increased risk for metabolic comorbidities such as diabetes mellitus or metabolic syndrome, but also for non-metabolic comorbidities such as neoplasms and liver disease. Therefore, the detection of incidentalomas may change the preoperative evaluation, requiring further investigations, such as magnetic resonance imaging (MRI) or biopsies, to exclude malignancy. It may even delay surgery until the diagnosis is clarified, affecting therapeutic planning. However, this does not change the quality of medical care, which will improve through careful analysis of the implications of these issues [20-22].

Our research on overweight and BS aims to identify and report the unique characteristics of obese patients with incidentalomas. All participants were BS candidates and were included in this study after acceptance for a surgical procedure at our specialized BS center. We performed a thorough analysis of this cohort of morbidly obese patients with incidentalomas and systematically present the results to contribute to a robust study that will help improve successful discharge management for this specific patient population.

## MATERIAL AND METHODS

### Type of study

This retrospective, observational study was conducted at a single medical center. We meticulously investigated the prevalence and characteristics of incidentalomas identified on preoperative abdominopelvic CT scans in 131 adult patients with obesity who underwent BS between January 2023 and December 2024.

Data were collected via a retrospective review of electronic medical records and detailed radiology reports to ensure the extraction of pertinent clinical and radiological information.

### Inclusion and exclusion criteria

The inclusion criteria mandated that all participants be adults with a BMI≥40 kg/m^2^ or≥35 kg/m^2^ with at least one comorbidity necessitating BS. Furthermore, all included individuals must have undergone standard preoperative abdominopelvic CT imaging as part of their routine clinical assessment.

Exclusion criteria were carefully defined to maintain data integrity and minimize potential confounders. Specifically, patients with incomplete or poor-quality preoperative imaging were excluded, as were those with a documented history of malignancy diagnosed prior to the abdominopelvic CT examination.

### Variables and statistical data

Key variables analyzed included patient demographics (age, sex) and the specific type of bariatric procedure performed. We also gathered comprehensive details on any incidentalomas discovered, noting their type, anatomical location, and the distinctive radiological characteristics described in the imaging reports.

The study's goal was to identify potential associations between incidentalomas and specific patient characteristics. We employed descriptive statistical analyses to summarize frequencies and percentages, alongside analytical statistical methods to explore potential correlations.

The study adhered to the principles of ethical research, ensuring patient confidentiality and compliance with institutional review board guidelines [23]. Approval was received from the Ethics Committee of the Grigore T. Popa University of Medicine and Pharmacy, Iasi.

## RESULTS

### Demographic profile of study participants

Of the 131 patients who were candidates for BS, our study identified 29 with incidentalomas on preoperative abdominopelvic CT scans, representing an overall incidence of 22.14%. Among these patients, 26 (89.7%) were women and only three (10.3%) were men, reflecting the female predominance in BS. The mean age was 39.0 ± 11.6 years (range 19-61 years), with a mean preoperative BMI of 38.5 ± 4.6 kg/m^2^ (Table 1).

**Table 1:** Baseline demographic and anthropometric characteristics

Characteristic	Mean ± SD / *n* (%)	Median (IQR)	Range
Age (years)	39.0 ± 11.6	39 (30–48)	19–61
Sex - Female, *n* (%)	26 (89.7%)	N/A	N/A
Sex - Male, *n* (%)	3 (10.3%)	N/A	N/A
Height (cm)	165.8 ± 7.3	165 (160–172)	155–180
Weight (kg)	105.8 ± 14.4	105 (96–119)	76–132
BMI (kg/m^2^)	38.5 ± 4.6	38.0 (36.0–40.2)	29.7–50.3
Abdominal circumference (cm)	112.1 ± 11.5	116 (104–121)	89–132
Abdominal wall thickness (mm)	52.7 ± 14.1	50 (45–59)	29–88

*n*, numbers; N/A, not applicable

### Bariatric procedure distribution

Of the 29 patients with incidentalomas, 20 (69.0%) underwent SG, and 9 (31.0%) underwent RYGB. The incidence of incidentalomas did not differ significantly between the two procedures: 21.74% for SG and 23.08% for RYGB (*P* = 1.000).

### Type and location of incidentalomas

A total of 64 incidentalomas were identified in the 29 patients, with a mean of 2.2 ± 1.3 incidentalomas per patient (range 1–5). The most frequent types are described in Table 2.

**Table 2:** Incidentaloma types and frequencies

Incidentaloma Type	*n*	Percentage (%)
**Hernia**	9	14.1%
**Hepatomegaly**	9	14.1%
**Hepatic steatosis**	9	14.1%
**Simple cyst**	7	10.9%
**Thymic remnant**	4	6.2%

*n*, numbers

The most frequent incidentalomas were liver-related, including steatosis and hepatomegaly, followed by hernias, simple cysts, thymic remnants, and findings involving the reproductive organs. When grouped by organ systems, the hepatic system was the most affected, accounting for 31.2% of incidentalomas. This was followed by other findings (18.8%), gynecologic (15.6%), abdominal wall/hernias (14.1%), digestive (12.5%), and renal (7.8%).

### Statistically significant correlations

**Figure 2:**
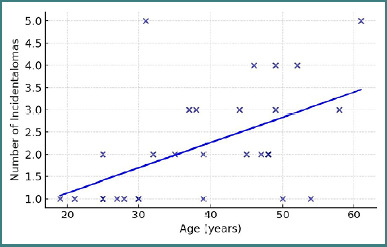
Correlation between age and number of incidentalomas per patient

The statistical analysis identified a significant positive correlation between age and number of incidentalomas per patient (r = 0.52, *P* = 0.004) (Figure 2). This result suggests that older patients had a higher probability of presenting multiple incidentalomas.

The ANOVA test confirmed statistically significant differences between age groups (F = 5.19, *P* = 0.006; Table 3).

**Table 3:** Distribution of the number of incidentalomas by age groups

Age groups	Incidentaloma/patient
≤30 years	1.11 ± 0.33
31–40 years	2.57 ± 1.27
41–50 years	2.56 ± 1.01
>50 years	3.25 ± 1.71

### Comorbidities

Twenty patients (69.0%) had comorbidities, while nine (31.0%) had none. Comparison between groups showed that patients with comorbidities were significantly older (42.1 ± years vs. 32.3 ± years, *P* = 0.034), with no significant differences regarding BMI, abdominal circumference, or number of incidentalomas.

### BMI category analysis

Distribution by BMI category demonstrated a progressive increase in the number of incidentalomas with greater degrees of obesity:

- 30–35 kg/m^2^: 1.50 incidentalomas/patient (6 patients);
- 35–40 kg/m^2^: 2.00 incidentalomas/patient (13 patients);
- 40–45 kg/m^2^: 2.86 incidentalomas/patient (7 patients);
- >45 kg/m^2^: 3.00 incidentalomas/patient (3 patients).

## DISCUSSION

Our research provides valuable insights into the prevalence and characteristics of incidentalomas in obese patients undergoing preoperative abdominopelvic CT evaluation for BS. Our findings of a 22.14% overall incidence of incidentalomas align with previous literature reporting incidental findings in 15–30% of diagnostic imaging studies and 20–40% of CT examinations. This relatively high prevalence underscores the clinical significance of systematic imaging evaluation in this population [24].

The most striking finding of our study is the strong positive correlation between age and the number of incidentalomas per patient (r = 0.52, *P* = 0.004). This relationship demonstrates statistically significant differences across age groups (p = 0.006), with patients >50 years presenting a mean of 3.25 ± 1.71 incidentalomas compared to 1.11 ± 0.33 in patients ≤30 years. These findings are consistent with the established literature, which shows that the prevalence of incidentalomas increases substantially with advancing age. For example, adrenal incidentalomas have been reported to affect approximately 3% of individuals aged 50 years, rising to 10% in those >70 years. The age-related increase likely reflects cumulative exposure to various pathophysiological processes, hormonal changes, and degenerative conditions over time [25]. Thus, age showed a more consistent positive correlation with the presence of multiple incidentalomas, suggesting that older patients are more likely to accumulate asymptomatic lesions over time. This observation is clinically relevant, as bariatric candidates are increasingly older, and age may be an independent predictor of incidental findings.

Also, our study revealed that hepatic involvement is the most common organ system affected (31.2% of all incidentalomas), with hepatomegaly, hepatic steatosis, and simple hepatic cysts as the predominant findings. The high prevalence of hepatic steatosis (14.1% of all incidentalomas) is particularly relevant in the context of BS candidates, as non-alcoholic fatty liver disease (NAFLD) has been reported in 80-90% of morbidly obese patients undergoing weight loss surgery. This finding supports the established association between obesity and hepatic steatosis, in which CT imaging demonstrates high specificity (90–100%) for detecting moderate to severe steatosis with liver attenuation values <48 HU [26-28].

Relatedly, the therapeutic implications are significant, as BS has been shown to effectively improve NAFLD outcomes, with studies reporting improvement in steatosis in 88% of patients and in steatohepatitis in 59% following surgical intervention. This suggests that the incidental detection of hepatic steatosis on preoperative CT may actually identify patients who will derive additional metabolic benefits from BS beyond weight loss alone [28,29].

Moreover, the high frequency of hernias (14.1% of all incidentalomas) in our cohort reflects the established relationship between obesity and increased intra-abdominal pressure, which can lead to abdominal wall defects. Previous studies have documented incidental hernia discovery rates of 5.3% during abdominal contouring procedures in post-BS patients, with most hernias located in paraumbilical/umbilical (46.7%) or epigastric (37.8%) regions. The detection of these hernias during preoperative evaluation enables surgical planning and, if feasible, simultaneous repair during the bariatric procedure, optimizing patient outcomes and reducing the need for subsequent interventions [30].

Interestingly, our study did not demonstrate a significant correlation between BMI and the number of incidentalomas (r = 0.28, *P* = 0.148), despite previous studies suggesting that obese subjects have a fivefold greater risk of incidental abnormalities on imaging. This finding may be attributed to the relatively narrow BMI range in our BS cohort (mean BMI 38.5 ± 4.6 kg/m^2^), where all patients met criteria for severe obesity. However, we observed a trend toward increasing incidentalomas with higher BMI categories, suggesting that the relationship may become more apparent across a broader BMI spectrum [31].

The absence of significant differences in incidentaloma prevalence between SG (21.74%) and RYGB (23.08%) procedures (*P* = 1.000) indicates that the choice of bariatric procedure does not influence the likelihood of incidental findings. This finding supports current clinical practice, where procedure selection is based on patient-specific factors and contraindications rather than concerns about differential incidentaloma detection rates.

Also, the high incidence of incidentalomas in our study population necessitates standardized approaches to preoperative imaging interpretation and follow-up protocols. While most incidental findings in healthy volunteers are benign, approximately 12.8% are clinically significant and require further evaluation or intervention. The age-related increase in incidentalomas suggests that older BS candidates may benefit from more comprehensive imaging evaluation and multidisciplinary consultation [31].

From a surgical perspective, the distribution of incidentalomas in sites such as the liver, ovaries, and abdominal wall (e.g., hernias) has implications for intraoperative decision-making. Surgeons should be aware of these common locations, as findings such as hiatal hernias or unexpected abdominal wall defects may alter the operative strategy. Additionally, reproductive or renal lesions may necessitate referral for further evaluation, ensuring comprehensive patient care beyond BS alone.

The presence of thymic remnants in 6.2% of our patients, primarily in younger individuals, aligns with the normal age-related involution process of the thymus gland. Studies have shown that thymic fatty replacement occurs gradually after age 30, but soft tissue components may persist in up to 9% of individuals >60 years. The identification of thymic remnants as incidentalomas in BS candidates is clinically relevant as these findings may be mistaken for pathological masses without appropriate age-related interpretation [32].

However, our single-center study with a relatively small sample size (131 BS candidates, 29 patients with incidentalomas) limits the generalizability of the findings. Additionally, the retrospective design precluded systematic follow-up of incidental findings to determine their long-term clinical significance. Future prospective multicenter studies with larger cohorts and standardized imaging protocols would provide more robust evidence regarding the optimal management of incidentalomas in BS candidates.

Overall, these findings support the role of CT as a valuable tool in preoperative assessment, not only for anatomical evaluation but also for uncovering clinically silent conditions. The relatively high rate of incidentalomas emphasizes the need for multidisciplinary collaboration in the management of bariatric patients, involving hepatologists, gynecologists, and general surgeons when necessary. Future studies with larger cohorts are warranted to further characterize the clinical significance of these incidental findings and to determine whether they impact long-term surgical outcomes.

## CONCLUSION

In conclusion, our study demonstrates that incidentalomas are highly prevalent in obese patients undergoing preoperative CT evaluation for BS, with age serving as the primary predictive factor for multiple findings. The predominance of hepatic involvement reflects the metabolic consequences of obesity and suggests potential additional benefits from surgical intervention.

Also, these findings support the implementation of systematic preoperative imaging evaluation protocols, with particular attention to age-related risk stratification and multidisciplinary management of significant incidental findings. The identification of incidentalomas should not delay BS but rather inform comprehensive perioperative care planning to optimize patient outcomes.
